# Isolated Mammillary Bodies Damage—An Atypical Presentation of Wernicke Syndrome

**DOI:** 10.3390/bs8110104

**Published:** 2018-11-12

**Authors:** Samar A. Abbas, Halim Abboud, Moussa A. Chalah, Chadi Sabbagh, Samar S. Ayache

**Affiliations:** 1 Department of Neurology, Hôtel-Dieu de France Hospital, Faculty of Medicine, Saint-Joseph University, Beirut 1104-2020, Lebanon; samar.abbas@net.usj.edu.lb (S.A.A.); halim.abboud@usj.edu.lb (H.A.); 2 EA 4391, Excitabilité Nerveuse et Thérapeutique, Université Paris-Est-Créteil, 94010 Créteil, France; samarayache@gmail.com; 3 Service de Physiologie—Explorations Fonctionnelles, Hôpital Henri Mondor, Assistance Publique—Hôpitaux de Paris, 94010 Créteil, France; 4 Department of Emergency Medicine, Hôtel-Dieu de France Hospital, Faculty of Medicine, Saint-Joseph University, Beirut 1104-2020, Lebanon; Chadi.sabbagh@usj.edu.lb

**Keywords:** brain MRI, Wernicke encephalopathy, mammillary bodies, atypical Wernicke encephalopathy, memory loss, amnesia

## Abstract

We report atypical magnetic resonance imaging (MRI) lesions in a case of Wernicke encephalopathy. The patient presented with isolated anterograde amnesia following a partial colectomy complicated by peritonitis. Fluid-attenuated inversion recovery and T2 MRI sequences were normal. However, bilateral contrast enhancement of mammillary bodies was shown on T1 gadolinium-enhanced sequences. Blood tests revealed thiamine deficiency. The diagnosis of Wernicke encephalopathy was made and thiamine supplementation was given, resulting in complete recovery of the memory functions.

## 1. Introduction

Wernicke Encephalopathy (WE) is a neurologic complication resulting from vitamin B1 (i.e., thiamine) deficiency. In 1881, Carl Wernicke reported this encephalopathy for the first time and described it as a clinical triad of altered consciousness (ranging from mild disorientation to coma), ophthalmoplegia, and gait ataxia. Since then, this classic triad has been described in only 16–38% of patients [1].

Ever since, WE has been more commonly associated with alcohol consumption, and there is always a delay in suggesting this encephalopathy in nonalcoholic patients, particularly when they present with altered mental status without the characteristic triad. The importance of an early diagnosis resides in the fact that emergent treatment by thiamine supplementation can prevent death and serious morbidity. WE diagnosis requires a combination of a high level of clinical suspicion and particular magnetic resonance imaging (MRI) findings. In an attempt to raise awareness on the atypical presentation of this entity, we hereby report a case of nonalcoholic WE.

## 2. Case Presentation

A 55-year-old woman presented to our emergency department because of a four-week history of memory loss, which was of insidious onset and progressive worsening. Her past medical history was relevant for Crohn’s disease and partial colectomy. The latter took place two months prior to presentation and was complicated by peritonitis, which was successfully treated with antibiotics. There was no history of alcohol consumption or vascular risk factors, and no family history of cognitive disturbances.

**Figure 1:**
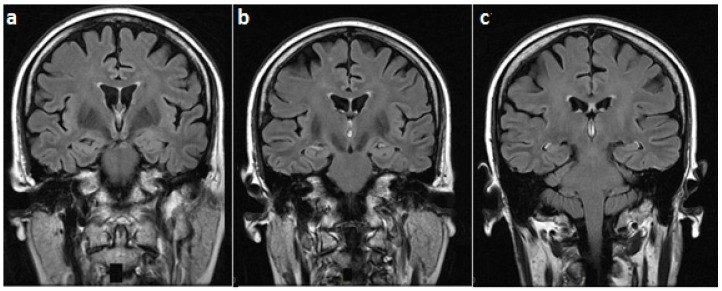
Fluid-attenuated inversion recovery brain magnetic resonance (MR) images (coronal view), at the level of (**a**) mammillary bodies, (**b**) third ventricle, and (**c**) midbrain, showing a normal magnetic resonance imaging (MRI) signal in these regions.

**Figure 2:**
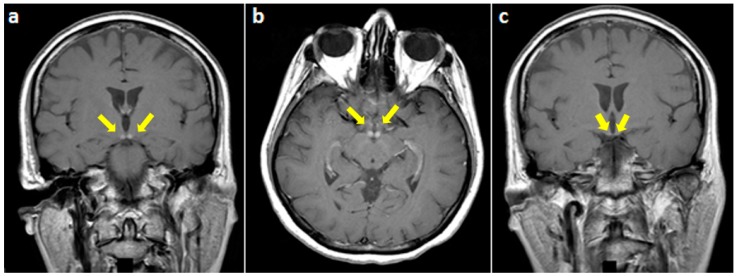
T1-weighted gadolinium-enhanced MRI at the level of mammillary bodies (yellow arrows) ((**a**) coronal and (**b**) axial views) and (**c**) the third ventricle showing an isolated enhancement of the former.

On examination, she was alert and cooperative, but disoriented to time and place, and unable to recall three objects on a mini-mental status exam. For the latter exam, the score was 18/30 on admission. Other cognitive domains were intact. In addition, cranial nerves as well as motor, sensory, and cerebellar functions were normal. The patient underwent brain MRI imaging which revealed normal T1, T2, and fluid-attenuated inversion recovery (FLAIR) sequences (Figure 1). However, T1 gadolinium-enhanced sequences demonstrated an isolated enhancement of the mammillary bodies (Figure 2).

Laboratory tests demonstrated an important decrease of blood thiamine levels (12.7 ng/mL (normal range: 21.3–81.9 ng/mL); obtained on a 2-mL sample of whole blood using High Performance Liquid Chromatography, Eurofins Biomnis, Lyon, France). Therefore, an intravenous thiamine supplementation was started with 1500 mg per day for three days (VITAMINE B1 STEROP solution containing 100 mg/2 mL, STEROP laboratories, Brussels, Belgium). The drug solution was diluted in 50 mL of sodium chloride 0.9 % and administered over 30 min.

The patient showed a partial memory improvement three days later. She was discharged home on oral thiamine therapy for one month (100 mg per os per day). At that time, she completely recovered and regained her normal eating habits (i.e., diet including meat, dairy products, fruits, vegetables, and other sources of carbohydrates).

The local ethics committee approved case publication, and written informed consent was obtained from the patient.

## 3. Discussion

Our patient presented an isolated anterograde amnesia following thiamine deficiency in a nonalcoholic context. In the absence of chronic alcohol consumption, numerous factors have been incriminated in the occurrence of this condition. In fact, a lack of thiamine has been found in several situations such as malnutrition, hyperemesis gravidarum, parenteral nutrition, gastrointestinal disorders, cancer, chemotherapy, thyrotoxicosis, surgeries, and infectious and systemic diseases [2]. In the current case, Crohn’s disease, colectomy, and peritonitis might have led to malnutrition and malabsorption, resulting in decreased thiamine levels.

In the absence of the classical triad of altered consciousness, ocular dysfunction, and ataxia, the diagnosis of WE is usually challenging for the medical community. Atypical presentation of WE is often described in nonalcoholic patients where a single element of the triad is usually found (i.e., delirium, diplopia, or gait ataxia). Cases of isolated memory loss, seizures, or lower limb weakness have also been reported [3]. In line with previous studies, our patient presented isolated anterograde amnesia; her consciousness and other cognitive domains were preserved.

**Figure 3:**
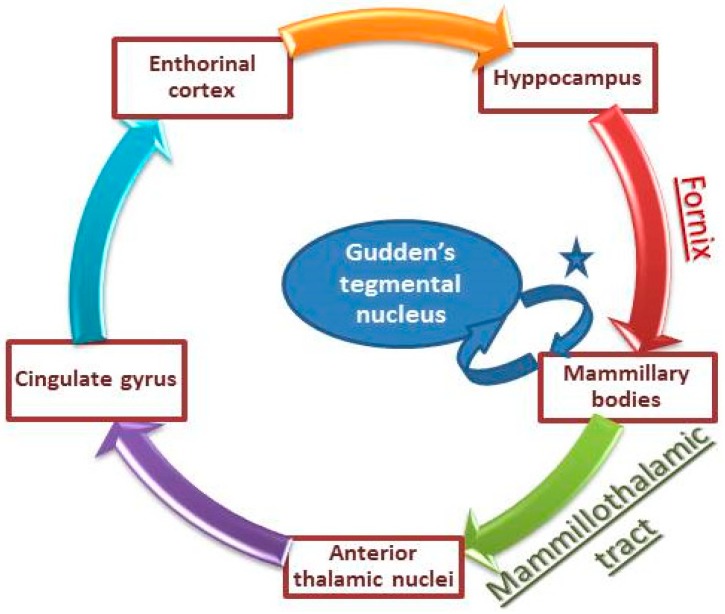
Illustration of Papez circuits involved in memory consolidation. * mammillary peduncle and mammillotegmental tract (adapted from Male and Zand [5]).

In order to explain our patient symptoms, it is important to mention that mammillary bodies play a pivotal role in memory formation and emotion processing. In fact, mammillary bodies are part of the Papez circuits and they help in conveying hippocampal inputs to the thalamus (anterodorsal and mediodorsal nuclei) through the mammillo-thalamic tract [4]. Although they have long been considered simple relay stations, recent human studies have shown that isolated damage to mammillary bodies could produce severe memory impairment. Such impairment was found to be global [5], or restricted to anterograde memory loss [6]. Given these data, a non-hippocampal pathway has been recently suggested as a crucial part of the memory network. This new pathway involves the Gudden’s tegmental nucleus—small cell groups in the caudal part of the midbrain—which has reciprocal connections with mammillary bodies, providing a non-hippocampal circuit for memory processing (Figure 3) [5].

Concerning MRI data, hyperintensities on T2 and FLAIR sequences have been observed in typical structures. The latter include the medial thalami, mammillary bodies, tectal plate, and peri-aqueductal area [7,8]. In typical WE, bilateral and symmetric involvement of the aforementioned areas constitutes a key MRI feature. However, atypical MRI findings have been mostly reported in nonalcoholic WE, and consist of T2 and FLAIR signal changes (hyperintensities) located in the cerebellum, cranial nerves nuclei, red nuclei, splenium, caudate, putamen, and/or cerebral cortex (fronto-parietal, peri-rolandic) [9,10,11,12]. Interestingly, isolated involvement of the mammillary bodies has rarely been reported in the literature and even less in nonalcoholic WE [7,10,13]. Moreover, in two studies conducted by Zuccoli et al. and Weidauer et al., contrast enhancement in typical areas of WE has been found in the absence of abnormalities on T2 and FLAIR sequences [11,14]. This pattern was observed in only a few cases; most of them were alcoholic. Interestingly, it was frequently seen at the level of mammillary bodies [10].

From a pathophysiological perspective, several mechanisms have been suggested to underlie the generation of WE [15]. In fact, the stored form of thiamine plays a key role as a cofactor for several enzymes that take part in the Krebs cycle and the pentose phosphate pathway; the latter are crucial for aerobic cellular respiration, energy production, and nucleic acid synthesis [15]. Therefore, thiamine depletion might result in several metabolic alterations, energy deficits, and cerebral lactic acidosis [15]. Such metabolic alterations result in the accumulation of toxic intermediates (i.e., glutamate), an increase in intracellular calcium concentration, and subsequently the death of glial cells and neurons. The latter would be no longer able to control ionic gradients across the cell membrane, leading to cytotoxic edema. Physical and/or chemical-mediated alterations would also occur at the level of endothelial cells, resulting in a dysfunction of the blood-brain barrier and thus the generation of vasogenic edema.

Finally, it is worth noting that the present case is limited by the absence of post-treatment MRI assessment. This could have allowed a comparison of neuroimaging data, as well as a confirmation of the link between thiamine deficiency and changes in the mammillary bodies.

## 4. Conclusions

To conclude, this case raises awareness on nonalcoholic WE, highlights the role of mammillary bodies in memory processing, and demonstrates the utility of gadolinium-enhanced T1 sequences in the diagnosis of WE.

In nonalcoholic patients, the main barriers to diagnose WE are the absence of the classic triad. Nevertheless, high suspicion of WE should be raised in all clinical settings that predispose to altered thiamine intake and/or absorption leading to serious deficiency. Early diagnosis (i.e., measurement of blood thiamine levels) and prompt thiamine supplementation are mandatory in order to prevent irreversible consequences (Korsakoff’s syndrome). Negative T2, FLAIR, and diffusion-weighted imaging sequences do not rule out the diagnosis, and T1 enhanced sequences should be included in the diagnostic armamentarium of this condition.
